# Supplementary material for: Impact of Dietitian-Guided Individualized Nutrition (DGIN) on ICU Outcomes in Critically Ill Patients: A Retrospective Cohort Study in Taiwan
Source: Nutrients. 2025 Sep 18;17(18):2995. doi: 10.3390/nu17182995 (PMC12472341; doi:10.3390/nu17182995)
Supplement: Supplementary file 1 [file nutrients-17-02995-s001.zip › nutrients-3829119-supplementary.pdf]

Supplementary Table S1 Baseline characteristics of patients after propensity score matching

Group	SC (*n* = 968)	DGIN (*n* = 968)	p value
Sex, Female/Male (%)	377 (38.9)/591 (61.1)	376 (38.8)/592 (61.2)	0.963
Age, years	66.1 ± 16.4	66.0 ± 16.5	0.933
Height, cm	161.1 ± 9.7	161.1 ± 9.6	0.663
Patient Type, *n* (%)			0.518
- Medical	577 (59.6)	563 (58.2)	
- Surgical	391 (40.4)	405 (41.8)	
Cancer diagnosis, *n* (%)	111 (11.5)	105 (10.8)	0.665
**Initial primary data**			
Weight, kg	61.6 ± 13.9	62.3 ± 15.7	0.489
BMI, kg/m^2	23.8 ± 6.9	24.0 ± 6.2	0.261
APACHE II score	17.9 ± 6.9	17.7 ± 7.3	0.663
Energy intake, kcal/kg/day	14.0 ± 10.7	13.7 ± 10.6	0.520
Protein intake, g/kg/day	0.5 ± 0.4	0.5 ± 0.4	0.461
**Initial laboratory values**			
Albumin, g/dL	3.1 ± 0.6	3.2 ± 0.7	0.215
Prealbumin, mg/dL	13.0 ± 7.2	12.9 ± 7.7	0.871
Hemoglobin, g/dL	10.2 ± 2.1	9.9 ± 2.0	0.086
BUN, mg/dL	35.7 ± 36.6	37.4 ± 35.6	0.093
Creatinine, mg/dL	1.9 ± 2.1	1.9 ± 2.2	0.751
Potassium, mmol/L	3.8 ± 0.8	3.9 ± 0.7	0.010
Calcium, mg/dL	8.2 ± 0.8	8.2 ± 0.8	0.944
Magnesium, mg/dL	2.1 ± 0.4	2.1 ± 0.4	0.942
Phosphorus, mg/dL	4.0 ± 1.9	4.1 ± 2.0	0.564
C-reactive protein, mg/L	6.1 ± 6.8	6.3 ± 7.2	0.721

Some variables have missing data; values are presented based on available cases. Continuous variables are presented as mean ± standard deviation (SD), and categorical variables are presented as number (percentage). p values for categorical variables were calculated using the Pearson χ^2 test; for continuous variables, the independent t-test was used. $p < 0.05$ and $p < 0.01$ are considered statistically significant and highly significant, respectively.

Supplementary Table S2 Multivariate Analysis of Clinical Outcomes Between dietitian-guided individualized nutrition (DGIN) and standard care (SC) Groups after propensity score matching

Variable	SC (*n* = 968)	DGIN (*n* = 968)	p value	OR (95% C.I.)†	p value
**Energy Intake, kcal/kg/day**					
At ICU admission	14.0 ± 10.7	13.7 ± 10.6	0.520	—	0.000
At ICU discharge	19.1 ± 9.9	18.4 ± 10.2	0.095	—	0.000
**Protein Intake, g/kg/day**					
At ICU admission	0.5 ± 0.4	0.5 ± 0.4	0.461	—	0.000
At ICU discharge	0.8 ± 0.4	0.7 ± 0.4	0.041	—	0.000
Body Weight Change, kg	0.0 ± 3.6	0.2 ± 3.9	0.593	—	0.000
Post-ICU BMI, kg/m²	23.9 ± 7.2	24.1 ± 6.1	0.117	—	0.000
APACHE II Score (post-ICU)	12.0 ± 7.3	12.1 ± 7.4	0.800	—	0.000
**Laboratory Parameters**					
Albumin, g/dL	3.2 ± 0.6	3.2 ± 0.6	0.108	—	0.000
Prealbumin, mg/dL	16.9 ± 8.2	14.2 ± 7.9	0.034	—	0.000
Hemoglobin, g/dL	10.2 ± 2.1	9.9 ± 2.1	0.019	—	0.000
BUN, mg/dL	35.1 ± 33.6	37.8 ± 36.7	0.147	—	0.000
Creatinine, mg/dL	1.9 ± 2.1	1.9 ± 2.2	0.674	—	0.000
Potassium, mmol/L	3.9 ± 0.8	4.0 ± 0.7	0.082	—	0.000
Calcium, mg/dL	8.3 ± 0.8	8.3 ± 0.9	0.739	—	0.000
Magnesium, mg/dL	2.1 ± 0.5	2.1 ± 0.4	0.552	—	0.000
Phosphorus, mg/dL	4.0 ± 2.0	4.0 ± 1.9	0.376	—	0.000
C-reactive protein, mg/L	5.4 ± 6.5	5.5 ± 6.7	0.573	—	0.000
Clinical Outcomes					
**In-hospital mortality (%)**	20.5% (198/968)	20.6% (199/968)	0.955	1.05 (0.79-1.40)	0.123
14-day readmission (%)	11.0% (73/664)	25.2% (77/306)	<0.001**	2.42 (1.61-3.63)	<0.001
30-day readmission (%)	14.3% (84/588)	30.6% (70/229)	<0.001**	3.05 (2.01-4.62)	<0.001
**ICU Length of Stay, days**	8.1 ± 6.8	7.4 ± 7.6	<0.001	—	0.000

† Adjusted for BMI, APACHE II score, energy delivery, protein intake, albumin, and potassium. Continuous variables are presented as mean ± standard deviation (SD). p values were calculated using the independent t-test or Mann–Whitney U test, as appropriate. Categorical variables are shown as *n* (%) and compared using the Pearson χ^2 test. Odds ratios (ORs) with 95% confidence intervals (CIs) were calculated using multivariate logistic regression.
